# Genome-Wide Transcriptome and Metabolome Analyses Provide Novel Insights and Suggest a Sex-Specific Response to Heat Stress in Pigs

**DOI:** 10.3390/genes11050540

**Published:** 2020-05-11

**Authors:** Krishnamoorthy Srikanth, Jong-Eun Park, Sang Yun Ji, Ki Hyun Kim, Yoo Kyung Lee, Himansu Kumar, Minji Kim, Youl Chang Baek, Hana Kim, Gul-Won Jang, Bong-Hwan Choi, Sung Dae Lee

**Affiliations:** 1 Amimal Genomics and Bioinformatics Division, National Institute of Animal Science, RDA, Wanju 55365, Korea; kris87@korea.kr (K.S.); jepark0105@korea.kr (J.-E.P.); himansu@korea.kr (H.K.); hanakim0307@gmail.com (H.K.); kwchang@korea.kr (G.-W.J.); bhchoi@korea.kr (B.-H.C.); 2 Animal Nutrition and Physiology Team, National Institute of Animal Science, RDA, Wanju 55365, Korea; syjee@korea.kr (S.Y.J.); kihyun@korea.kr (K.H.K.); yoo3930@korea.kr (Y.K.L.); mjkim00@korea.kr (M.K.); chang4747@korea.kr (Y.C.B.)

**Keywords:** heat stress, pig, Duroc, RNA-Seq, NMR, metabolome

## Abstract

Heat stress (HS) negatively impacts pig production and swine health. Therefore, to understand the genetic and metabolic responses of pigs to HS, we used RNA-Seq and high resolution magic angle spinning (HR-MAS) NMR analyses to compare the transcriptomes and metabolomes of Duroc pigs (*n* = 6, 3 barrows and 3 gilts) exposed to heat stress (33 °C and 60% RH) with a control group (25 °C and 60% RH). HS resulted in the differential expression of 552 (236 up, 316 down) and 879 (540 up, 339 down) genes and significant enrichment of 30 and 31 plasma metabolites in female and male pigs, respectively. Apoptosis, response to heat, Toll-like receptor signaling and oxidative stress were enriched among the up-regulated genes, while negative regulation of the immune response, ATP synthesis and the ribosomal pathway were enriched among down-regulated genes. Twelve and ten metabolic pathways were found to be enriched (among them, four metabolic pathways, including arginine and proline metabolism, and three metabolic pathways, including pantothenate and CoA biosynthesis), overlapping between the transcriptome and metabolome analyses in the female and male group respectively. The limited overlap between pathways enriched with differentially expressed genes and enriched plasma metabolites between the sexes suggests a sex-specific response to HS in pigs.

## 1. Introduction

Heat stress affects animal husbandry worldwide and is a major environmental factor that affects animal health and production [1,2]. The increasing environmental temperature due to global warming will particularly affect pigs due to their lack of functional sweat glands, which would have helped in endogenous heat dissipation [3,4]. Added to this, the thick subcutaneous adipose tissue in the pig, impedes effective radiant heat loss [5]. Moreover the increase in metabolic rate due to rapid lean tissue accretion increases endogenous heat production, exacerbating the innate inability of porcine animals to tolerate heat [3,6,7,8,9]. The estimated annual economic loss due to heat stress for the swine industry in the US alone is nearly US $300 to $450 million [5,10,11], mainly due to increased mortality and morbidity, altered carcass composition, decreased feed efficiency, inconsistent growth, reduced fecundity and poor sow performance [6], so heat stress in a pressing issue for worldwide pig production.

Quiniou and Noblet [12] have shown that the growth rate is normally between 18 and 25 °C and that the thermoregulatory response in pigs is activated at about 25 °C. Animals respond to heat stress by regulating physiological and metabolic changes, such as redistribution of blood flow from the body core to the periphery, and by reducing feed intake [13] to reduce metabolic heat production [14]. This has a significant effect on animal health and productivity [3]. The heat stress mitigation options available at present include expensive heat abatement processes, such as spray or floor cooling and nutritional supplementation strategies. Since the response to heat stress varies within a population, due to genetic variations, selection for thermal tolerance could be a better alternative [1,15]. However traditional breeding programs have limitations due to the difficulties in collecting precise records of thermal states of individual animals. To overcome this, production traits that are correlated with heat stress are used for selecting animals with higher thermal tolerance [16]. They, however, mask the actual effects of heat stress; therefore, a precise and efficient method for identifying genetic tolerance to heat stress is necessary. Identifying genetic and metabolic markers that are sensitive to heat stress will be the most important step for managing heat stress in pigs. These markers could also be putative candidates for identifying genomic variants for improving heat tolerance in pigs.

Previous studies have shown that heat stress triggers a complex array of gene expression and metabolic changes in livestock, resulting in the modulation of a wide variety of pathways involved in the heat stress response, the inflammatory response, DNA damage repair, chaperone use, etc. [5,17,18,19]. Similarly, a variety of metabolites involved in carbohydrate, amino acid, fatty acid and amine metabolism are modulated, including glucose, lactate, alanine, cysteine, isoleucine, etc. [20,21,22,23]. However, these studies did not involve integrated transcriptome and metabolome profiling, which could lead to better understanding of the effect of heat stress on swine physiology, gene expression and metabolism, which will help in designing and implementing innovative strategies to limit the economic losses due to heat stress on pig farm profitability. Therefore, in this study, we exposed 3-month-old pigs to heat stress and performed transcriptome and metabolome analysis using RNA-Seq and high resolution magic angle spinning (HR-MAS) NMR (nuclear magnetic resonance) to understand the gene expression and metabolome perturbations in response to heat stress.

## 2. Materials and Methods 

### 2.1. Animals

The experiments were performed following the ethical guidelines laid down by the Institutional Animal Care and Use Committee (IACUC) of the National Institute of Animal Science (NIAS). The experimental procedures were reviewed and approved by IACUC of NIAS (NO 2017-1070). A total of 6 (3 barrows and 3 gilts) unrelated, 3-month-old Duroc pigs, weighing 56 ± 2.62 Kg, were used in this study. Animals were fed a corn and soybean meal based diet (Table 1), containing 18% crude protein, 4.9% crude fat, 4.6% crude fiber and 4.4% crude ash; the whole diet was formulated to provide 3450 Kcal/Kg. The animals had ad libitum access to feed and water. Water and feed intake were measured (Table S1). One-way analysis of variance (ANOVA) was performed in R to test for significance. Differences were considered to be significant at *p* < 0.05. Other than the typical heat stress responses, no significant differences in behavior were noted. 

### 2.2. Heat Stress Experimental Setup

The heat stress (HS) experiment was carried out in an environmental chamber which had controls for temperature and humidity in NIAS. After acclimation to the chamber for 3 days, the experiments were performed. The animals were first kept at 25 °C and 60% relative humidity for 24 h, and at the end, 10 mL of blood was drawn via venipuncture and 5 mL of blood was stored in a Tempus Blood RNA tube (Life Technlogies, Carlsbad, CA, USA) for RNAseq analysis and the rest of the blood was stored in a vacutainer tube containing anticoagulants (EDTAK2), for the metabolomic analysis. The same animals were then exposed to 33 °C temperature and 60% relative humidity for 24 h and blood was drawn and stored in the same way. 

### 2.3. RNA Isolation and Sequencing 

The RNA was isolated using TRIzol Reagent (Invitrogen, Carlsbad, CA, USA) following the manufacturer’s guidelines. The integrity and quality of the RNA was assessed using 2100 Bioanalyzer and RNA 6000 Nano LabChip Kit (Agilent Technologies, Palo Alto, CA, USA). Only samples with RIN values >8 were used for library construction. The concentration of RNA was determined using NanoDrop ND-1000 spectrophotometer (NanoDrop Technologies, Wilmington, DE, USA). The sequencing library was constructed using Illumina TruSeq RNA sample preparation kit (Illumina, San Diego, CA, USA) following processes previously described [16]. The sequencing was performed on an Illumina HiSeq 2000 sequencer. The raw reads are available for download from sequence read archive (SRA), NCBI under the accession number SUB7043048.

### 2.4. Sample Preperation and Metabolite Analysis with ^1^H-NMR

36 μL of Plasma was mixed with 4 μL of D_2_O (20 mM TSP-d4), and the sample was then transferred to a 4 mm NMR nanotub, and ^1^H-NMR spectra were obtained by high-resolution magic-angle-spinning (HR-MAS) NMR spectra using an Agilent 600 MHz NMR spectrometer (Agilent Technologies, Palo Alto, CA, USA) with a 4 mm gHX Nanoprobe. A Carr–Purcell–Meiboom–Gill (CPMG) pulse sequence was used to reduce signals generated by macromolecules and water. The ^1^H-NMR spectra were measured as described previously [24]. The TSP-d_4_ peak at 0.0 ppm was used as a reference for calibrating chemical shifts.

### 2.5. Data Analysis

#### 2.5.1. RNA-Seq Data

The processing of the data was previously described [25]. Briefly, after checking the quality of the raw reads using FastQC (version 0.11.5) [26] and trimming the adaptors and low-quality bases using TRIMMOMATIC (version 0.36) [27], the reads were aligned to the Pig reference genome (*Sus scrofa* 11.1) using HiSAT2 (version 2.05) [28]. The aligned reads were then counted using FeatureCounts (version 1.5.0) [29]. After correcting for batch and unknown effects using Svaseq [30], differential expression analysis was performed using DESeq2 [31]. Significant genes (FDR < 0.1) were identified, and functional enrichment analysis based on Gene Ontology (GO) under Biological Process and Molecular Function was performed with DAVID [32]. KEGG (Kyoto Encylopedia of Genes and Genomes (KEGG) pathway enrichment analysis was performed using ClueGO plugin [33] in cytoscape version 3.7.2 [34]. Goseq [35] was used for metabolic pathway enrichment analysis. 

#### 2.5.2. Metabolome Data 

The metabolite quantification was performed using Chenomx NMR suite 7.1 (Chenomx, Edmonton, Canada). The generated spectra were binned with a binning size of 0.001 ppm, and normalized to the total area, and binned data were aligned with the icoshift algorithm of MATLAB R^2^013b (MathWorks, Natick, MA, USA). Principle component analysis (PCA) and statistical analyses were performed using MetaboAnalyst 4.0 [36]. Only features that were detected in at least 50% of samples were used.

#### 2.5.3. Pathway Enrichment Analysis of Differentially-Enriched Metabolites 

Significantly differentially-enriched metabolites (*p* < 0.05) were subjected to KEGG pathway analyses using the Pathway analysis module in MetaboAnalyst 4.0 [36]. A combination of quantitative enrichment and topology analysis using only curated metabolic pathways from the KEGG database was used in the analyses. 

### 2.6. Real-Time PCR Validation

Real time reverse transcriptase PCR (qRT-PCR) was performed using gene-specific primes (Supplementary File S1). The PCR was performed on an ABI 7500 Real Time PCR system using Fast SYBR green master mix (Applied Biosystems, Foster City, CA, USA). A total of 18 genes (9 each from male and female differentially expressed gene (DEG) set) were analyzed. β-actin and GADPH (Glyceraldehyde-3-phosphate dehydrogenase) were used as endogenous controls. The stability of expression for each of those two genes was checked using GeNORM (https://genorm.cmgg.be/) against the same concentration of RNA from different samples. β-actin was the most stable and was used for normalizing the expression data of the target genes.

## 3. Results

### 3.1. Transcriptome Alignment, Mapping and Principle Component Analysis

**Figure 1:**
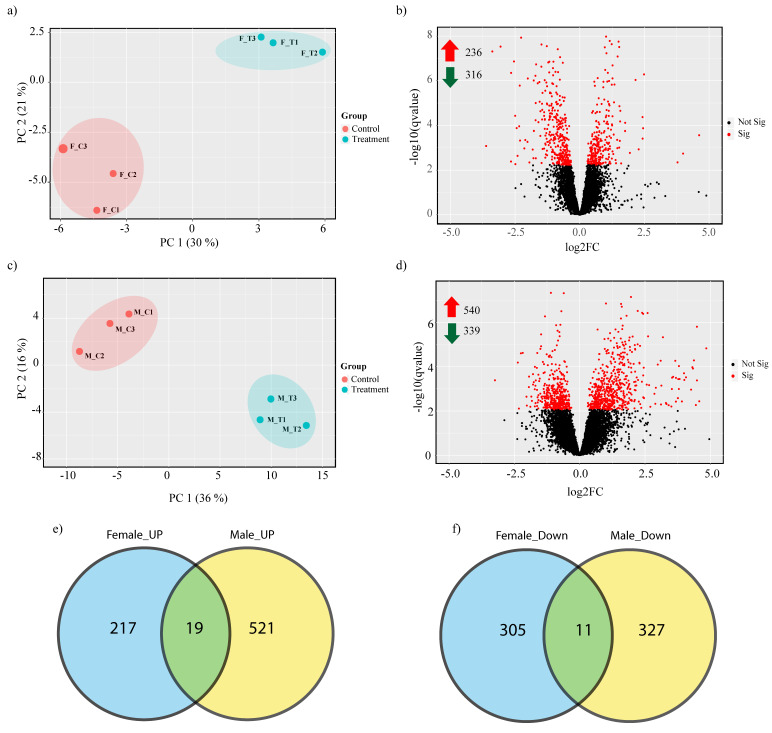
Summary of the transcriptome analysis: (**a**) PCA of female group samples; (**b**) volcano plot showing the number and distribution of significantly differentially expressed genes in the female group; (**c**) PCA of male samples; (**d**) volcano plot showing the number and distribution of significantly differentially expressed genes in the male group; Venn diagrams showing differentially expressed genes DEGs common between male and female groups: (**e**) up-regulated and (**f**) down-regulated.

We investigated the impact of heat stress on Duroc pigs using high throughput RNA-Seq analysis. A total of 423.3 million 100 bp Paired-End (PE) reads corresponding to an average of 35.2 million reads per individual was generated. After trimming for adapters and low-quality reads, 410.5 million reads remained. The reads were mapped to the *Sus scrofa* reference genome at an average alignment rate of 96.8% (File S2). The reads were mapped to a total of 19,283 genes. Principal component analysis (PCA) (Figure S1) suggested that sex had a large effect on transcriptome difference between the groups, so we decided to compare the heat stress effect on male and female pigs separately. PCA showed that 30% and 36% of the expression variation was due to heat stress in female and male pigs respectively (Figure 1a,c); however, considerable within group variation was also observed, confirming previous reports that the heat stress response varies within populations due to underlying genomic variation [1,15].

### 3.2. Porcine Transcriptome Response to Heat Stress

Heat stress resulted in 552 and 879 genes being significantly (FDR < 0.1) differentially expressed in male and female groups respectively. Out of those, 236 and 540 genes were up-regulated and 316 and 339 genes were down-regulated in female and male pigs respectively (Figure 1b,d, File S3). The genes modulated in response to heat stress were considerably different between the male and female groups, with only 19 up-regulated and 11 down-regulated genes being common between the groups (Figure 1e,f). Among the top up-regulated DEGs in the female group were *HIST2H2AA4 (*histone cluster 2H2A family member a4), *MYH4* (myosin heavy chain 4), *ABCA6* (ATP binding cassette subfamily A member 6), *HSF4* (heat shock factor 4) and *IL18* (interleukin 18), while top down-regulated DEGs included *CXCL10* (C-X-C motif chemokine 10), *IDO1* (indoleamine 2,3-dioxygenase 1), *HES4* (Hes family BHLH transcription factor 4) and *IFI6* (γ-interferon-inducible protein Ifi-16); in the male group the up-regulated genes included *DES* (Desmin), *RAG1* (recombination activating 1)*, LSAMP* (limbic system associated membrane protein), *CD1E* (CD1e molecule), *HSPG2* (heparin sulfate proteoglycan 2) and down-regulated genes included *CEBPE*(CCAAT/enhancer binding protein), *ZNF316* (Zinc Finger Protein 316)*, PXDC1*(PX domain containing 1) and *COX4I1* (cytochrome c oxidase subunit 4l 1). 

**Figure 2:**
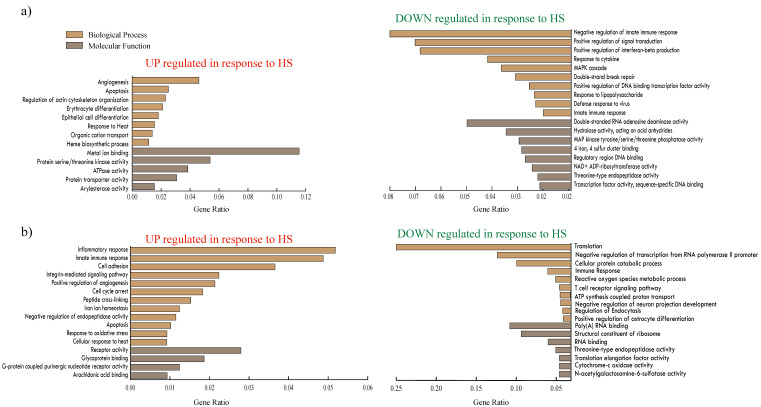
Gene Ontology analysis: (**a**) enriched GOs in female group; (**b**) enriched GOs in male group. The GO enrichment was performed under Biological Process and Molecular Functions categories.

**Figure 3:**
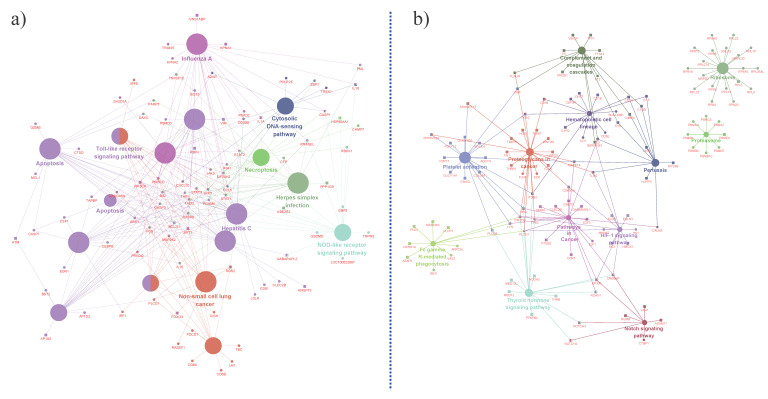
KEGG pathway enrichment analysis: (**a**) enriched KEGG pathways in female group; (**b**) enriched KEGG pathways in male group. Nodes are genes and edges represent pathways. Up-regulated genes are represented as rectangles and down-regulated genes are represented as hexagons.

Gene Ontology (GO) enrichment analysis of the significantly DEGs (FDR < 0.1) in the female group (Figure 2a) showed that among the up-regulated DEGs, “angiogenesis,” “apoptosis,” “response to heat,” “ heme biosynthetic process” and “ATPase activity” were enriched, whereas “negative regulation of innate immune response,” “positive regulation of signal transduction,” “response to cytokine,” “MAPK cascade,” “response to lipopolysaccharide,” “threonine-type endopeptidase activity,” etc., were enriched amongst the down-regulated genes. KEGG (Kyoto Encyclopedia of Genes and Genomes) pathway enrichment analysis of the female group DEGs (Figure 3a) showed that “apoptosis,” “non-small cell lung cancer,” “Toll-like receptor signaling pathway,” “necroptosis,” “NOD-like receptor signaling pathway” and “Influenza A” pathways were enriched. 

**Figure 4:**
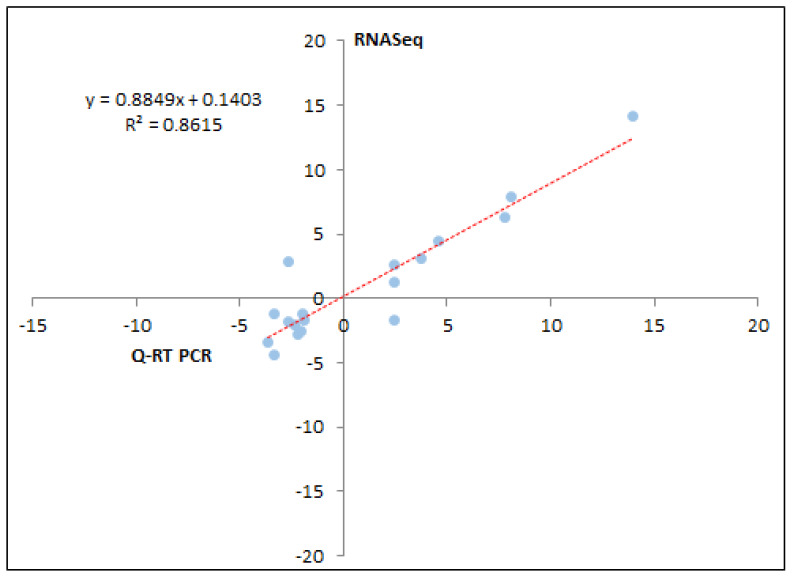
qRT-PCR validation of RNA-Seq results, using the top 10 DEGs from male and female groups respectively. The fold change from q-RT PCR was plotted on the *x*-axis, and the log_2_ fold change from the RNA-Seq analysis was plotted on the *y*-axis; and the correlation between the two methods is given in the figure.

In the male group, heat stress resulted in the up-regulation of genes functioning in “inflammatory response,” “innate immune response,” “cell adhesion,” “integrin-mediated signaling pathway,” “cell cycle arrest,” “positive regulation of angiogenesis,” “apoptosis,” “response to oxidative stress,” “cellular response to heat” and “glycoprotein binding.” Genes functioning in the “cellular protein catabolic process,” “regulation of endocytosis,” “negative regulation of astrocyte differentiation,” “cytochrome-c oxidase activity” and “N-acetylgalactosamine-6-sulfatase activity” were down-regulated (Figure 2b). Among KEGG pathways (Figure 3b), genes part of “complement and coagulation cascades,” “platelet activation,” “fc γ r-mediated phagocytosis,” “thyroid hormone signaling,” “pathways in cancer,” “proteoglycans in cancer,” and “Notch signaling” pathways were enriched. The top 10 DEGs from the female and male group were validated using q-RT PR analysis (quantitative Real-time PCR) (Figure 4); the correlation between RNA-Seq and q-RT PCR analyses was 0.861. 

### 3.3. Porcine Metabolome Response to Heat Stress

**Figure 5:**
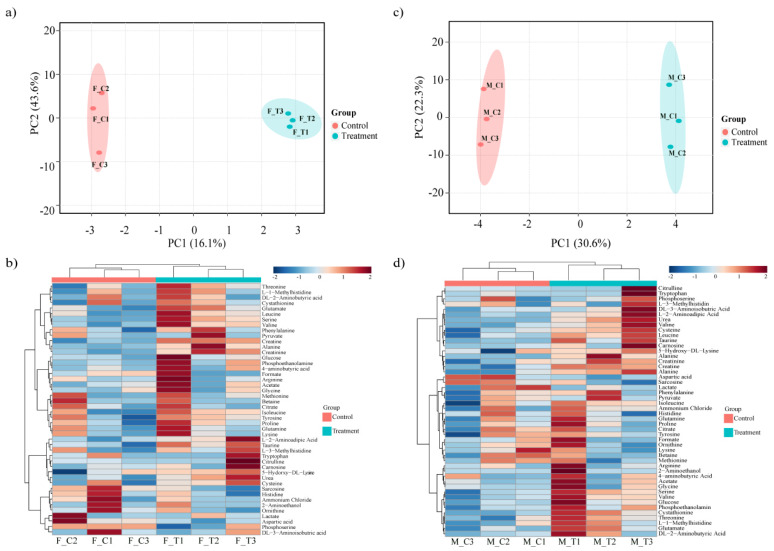
Metabolome analysis: (**a**) metabolic profiles of the female group visualized with principal component analysis; (**b**) heat map of all metabolites identified in the female group; (**c**) metabolic profiles of the male group visualized with principal component analysis; (**d**) heat map of all metabolites identified in the male group.

We detected and identified a total of 46 metabolites (Figure 5b,d, File S4). After normalizing the sample (quantile normalization) and scaling the data (auto scaling) in MetaboAnalyst 4.0 [36], PCA and a heatmap were generated. The overall metabolic profiles of the groups resolved well in PC1 (Figure 5a,c), and hierarchical clustering based on metabolite concentration showed that the control and heat stress group were well separated. However, PC2 showed that there was considerable within-group variation in both male and female pigs. The fold change was calculated as a ratio between the two groups’ means. A Wilcoxon rank-sum test was performed, and 30 and 31 metabolites were found to significantly differ (*p* < 0.05) between the control and heat stressed groups in female and male groups respectively (File S4).

In the female group, out of the 30 metabolites that significantly differed between the heat stress and control groups, the concentrations of 21 metabolites increased while nine metabolites decreased in concentration. The concentrations of tryptophan, arginine, pyruvate, alanine, acetate and lactate were higher, while DL-plus *allo*-*δ*-hydroxylysine, creatine, urea, 3-aminoisobutric acid, 4-aminobutyric acid and 3-methylhistidine were lower in the heat stress group (File S4). In the male group samples, 18 of the 31 significantly differing metabolites increased, among which where aspartic acid, pyruvate, betaine, creatine and lactate, while 13 metabolites, including tryptophan, carnosine, valine, alanine and creatine, were significantly reduced in the heat stress group compared to the control group (File S4).

### 3.4. Metabolic Pathways Enriched in Response to Heat Stress

**Figure 6:**
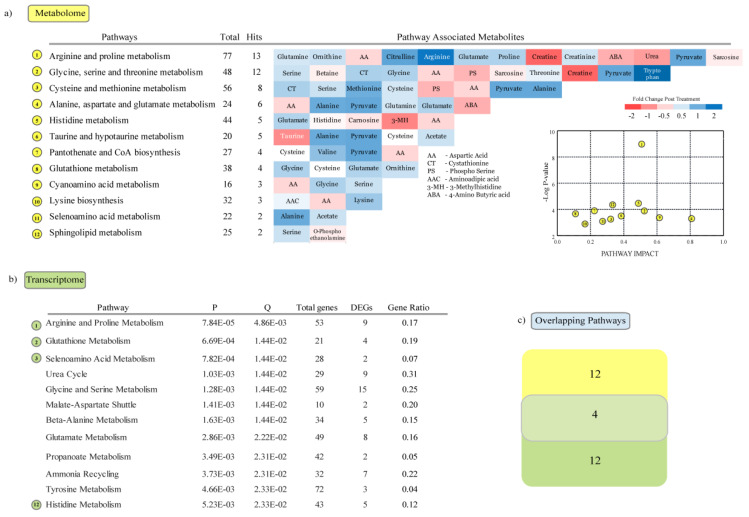
Metabolic pathways altered in the female group: (**a**) Metabolic pathway enrichment analyses of metabolites enriched in response to heat stress. (**b**) Metabolic pathway enrichment analyses of genes differentially expressed in response to heat stress. (**c**) Number of overlapping pathways enriched with DEGs and significantly differing metabolites in the heat stress group.

Pathway analysis was performed with metabolites that significantly differed between the heat stress and control group using the pathway enrichment module in MetaboAnalyst 4.0 [36]. Twelve pathways were significantly (*p* < 0.05) enriched in the female group (Figure 6a), including “arginine and proline metabolism,” “glycine, serine and threonine metabolism,” “cysteine and methionine metabolism,” “alanine, aspartate and glutamate metabolism” and “histidine metabolism”. We used goseq [35] to identify metabolic pathways in KEGG database that were significantly altered in the transcriptome, in response to heat stress. Among the twelve pathways significantly enriched (Figure 6b), four pathways were found to be commonly enriched with DEGs and DEMs (differentially enriched metabolites) (Figure 6c); these were “arginine and proline metabolism,” “glutathione metabolism,” “selenoamino acid metabolism” and “histidine metabolism.” Out of these, the first three pathways have been found to be enriched in response to oxidative stress induced by chronic HS in the pig [37,38,39]. 

**Figure 7:**
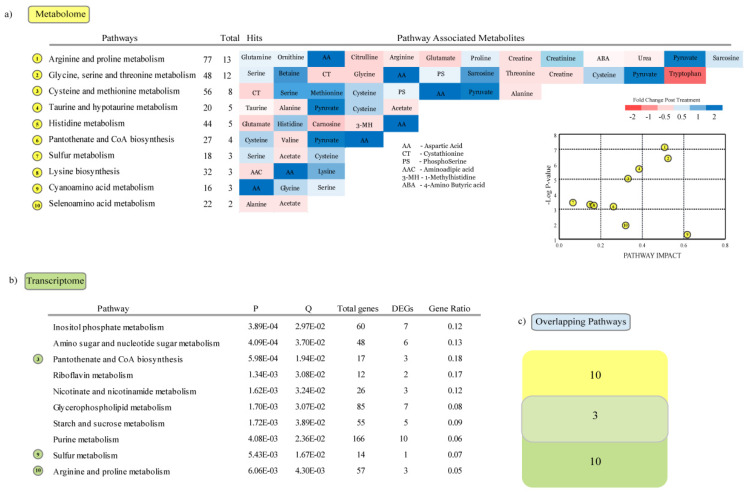
Metabolic pathways altered in the male group: (**a**) Metabolic pathway enrichment analyses of metabolites enriched in response to heat stress. (**b**) Metabolic pathway enrichment analyses of genes differentially expressed in response to heat stress. (**c**) Number of overlapping pathways enriched with DEGs and significantly differing metabolites in the heat stress group.

Among the male animals, 10 metabolic pathways were enriched with differently enriched metabolites, including “arginine and proline metabolism,” “glycine, serine and threonine metabolism,” “cysteine and methionine metabolism,” “taurine and hypotaurine metabolism” and “histidine metabolism” (Figure 7a). The metabolic pathways enriched with DEGs (Figure 7b) included “inositol phosphate metabolism,” “amino sugar and nucleotide sugar metabolism,” “pantothenate and COA biosynthesis” and “riboflavin metabolism.” Comparative analysis of the metabolic pathways enriched with DEMs and DEGs showed that three metabolic pathways were common (Figure 7c); those were “pantothenate and CoA biosynthesis,” “sulfur metabolism” and “arginine proline metabolism.” Pantothenate and CoA biosynthesis was previously found to be enriched in response to HS in the pig [5,20]. 

## 4. Discussion

Heat stress occurs when the rate of heat accumulation exceeds the rate of heat loss; i.e., when the animal is removed from the thermal comfort zone. HS causes the core body temperature of a pig to rise [40]. Temperature increase due to global warming will be detrimental to swine health and production. The global temperature has already been raising at an average of 0.13 °C over the past 50 years [41]. Pigs are particularly vulnerable due to their lack of functional sweat glands, and the thick layer of subcutaneous adipose tissue that they possess which impedes effective radiant heat loss [42]. Heat stress triggers an adaptive stress response by regulating a complex array of genes and metabolites [16,18,21]. In this study we found significant differences in water and feed intake post heat stress, and a significant difference in water intake post heat stress treatment between male and female pigs (Table S1).

Several studies have suggested that gender might have an effect on the heat stress response in pigs [40,43,44,45]. The sex-specific response to heat stress could be due to the difference in surface area to mass ratio or due to body compositional difference (lean meat to fat mass) which affects radiant heat loss [43]. Barrows are reported to generally have greater backfat thickness than gilts [46]. Though barrows consume more feed and gain body weight more rapidly, gilts deposit proportionally more muscle and less fat in their carcass [43,47]. This sex-specific difference in body composition could significantly alter the heat stress response, as it affects the ability of the animal to maintain the core body temperature through radiant heat loss. Moreover, several hormones, such as prostaglandins [48], endogenous opioids [48] and glucocorticoids have roles in the thermoregulatory mechanism [49]. Cortisol, a glucocorticoid, is the mostly widely used biomarker for detecting stress [50,51]. The concentration of cortisol is 15% higher in barrows than in gilts [52]. HS-induced increases in the concentrations of catecholamines and glucocorticoids have been observed in many species [53,54]; the effect of HS on immune cells is suggested to be dependent upon these hormones [55,56,57]. Similarly, Rudolph et al. [40], found that oxidative stress did not result in muscle injury in barrows, while previous studies in gilts reported that oxidative stress causes muscle injury [58,59,60,61]. This led them to suggest that muscle response to heat stress could be partially dependent upon sex of the pig, and that muscle tissues in males could be more resistant to heat stress than in females. The result of our study concurs with all these reports; our study shows that HS in pigs, as evidenced from transcriptome expression and metabolome concentration, is sexually dimorphic. 

### 4.1. Transcriptome Regulation in Response to Heat Stress

Transcriptome analysis showed that heat stress caused a profound shift in gene expression in the male group: 879 genes were found to be significantly differentially regulated in the male group, while 552 genes were DEs (differentially expressed) in the female group. Among the DEGs were several genes involved in Heat shock response. Heat shock proteins (HSPs) are expressed upon encountering stress [62,63]. Heat is proteotoxic and causes proteins to denature [64], HSPs act as chaperones in assisting in protein folding, thereby helping in avoiding protein aggregation [65] and maintaining cellular homeostasis [66]. *HSPA5* (heat shock 70 kDa protein 5)*, HSP90AA1* (heat shock protein 90 α family class a member 1) and *HSF4* (heat shock transcription factor 4) were up-regulated, in both male and female groups, while *FKBP5* (FKBP prolyl isomerase 5) was up-regulated only in the male group. *HSPA5* is a member of the HSP70 (heat shock protein 70) family of genes; it serves as an endoplasmic reticulum chaperone and as a sensor of protein misfolding [67]. *HSP90AA1* a member of the HSP90 family takes part in several cellular functions such as regulating protein activity, transport and also in activating different signaling pathways by forming complexes with steroid receptors [68]. *HSP90AA1* is essential for normal spermatogenesis in pigs [69], and regulation of *HSP90AA1* protects cells from heat shock [70]. *FKBP5* a member of immunophilin family, is a glucocorticoid receptor (GR)-regulating co-chaperone of heat shock protein 90, their expression are regulated by progestins and glucocorticoids [71], and are significantly correlated with plasma cortisol concentration in pig [72,73], suggesting that the male pigs were under considerable stress. 

Several co-chaperones and genes involved in cellular response to heat stress were also DEs; these included *HMOX1* (heme oxygenase 1)*, TRPM2* (transient receptor potential melastatin 2), which were up-regulated in the male group, *IL1A* (interleukin 1 α); up-regulated in both male and female groups, *SOD1* (superoxide dismutase 1) down-regulated in male group and TFEC (Transcription factor EC), DAXX (death domain associated protein), and TRPM2 which were down-regulated in the female group. *HMOX1* is an oxidative stress marker [74], and is up-regulated in response to oxidative stress [75]. *SOD1* catalyzes the removal of superoxide radicals that are generated due to biological oxidation [76]. *SOD* expression was found to be down-regulated in porcine skeletal muscle in response to long-term (3 days) heat stress [58]. In this study SOD expression was down-regulated in the male group following heat stress; this along with the increased expression of *HMOX1* suggests that the male group animals may have experienced significant oxidative stress following heat stress. Several genes involved in response to oxidative stress were up-regulated in the male group, including *TRPM2* [77], *LRRK2* [78], *MAPK14* [79] and *VNN1* [80]. In the female group *PRDX2* [81], *SLC7A11* [82] and *FOXO3* [83] which protects cells from ROX were up-regulated, while *TREX1* involved in cellular response to oxidative stress [84] and *SETX* (Senataxin) involved in defense against DNA damage due to oxidative stress [85] were down-regulated in the female group. 

Immune response is closely linked with response to heat stress. Immune stimulation could occur either due to the hyper thermic effect on immune cells or due to indirect effects such as the activation of heat shock factors, which are potent immune modulators and can stimulate both innate and adaptive immune response [86]. Heat stress triggered a massive innate immune response in the male group, with several genes including *CD14*, *BMX*, *S100A8*, *TLR4*, *MYD88*, *SRC*, *TLR2*, *SLEC5A*, *S100A9*, *PTK2B* and *VNN1* being up-regulated; interestingly in the female group, 13 genes involved in innate immunity were down-regulated; those included, *TRIM5*, *ISG20*, *RSAD2*, *DDX58*, *PML*, *TRIM25*, *TEC, IRF3*, *TRIM26*, *SH2D1A*, *ANXA1*, *FADD* and *JAK3.* In the male group, among the DEGs several inflammatory response genes were up-regulated; these included *CD14*, *CCR1*, *TSPAN2*, *TLR4*, *THBS1*, *PTGER3*, *MYD88*, *C5AR2*, *SELP*, *TLR2* and *CHI3L1*, while inflammatory response was not enriched amongst the DEGs in the female group. Anti-tumor immune response were also markedly enhanced due to heat stress, genes involved in proteoglycans in cancer, choline metabolism in cancer, pathways in cancer were up-regulated in both male and female groups, while apoptotic genes were only triggered in the female group; these included genes *PIK3CA*, *BCL2L1*, *AKT3* and *ATM.* Several studies have shown that heat stress triggers anti-tumor and apoptotic pathways possibly due to protein aggregation due to heat stress induced denaturation [16]. 

### 4.2. Metabolome Regulation in Response to Heat Stress

Metabolites are building blocks for growth and development; they are also key regulators and markers of animal health [87]. Blood metabolites are highly sensitive to environmental stress [88] and HS alters protein metabolism in a number of species [19,89,90,91]. Metabolome analysis showed that several plasma metabolites were enriched in response to HS in both male and female groups, this included, creatinine, histidine, lysine, methionine, ornithine, serine, proline and pyruvate, while 4-aminobutryic acid, Creatine, Taurine and Urea concentration was lower post HS. Plasma creatinine concentration has been found to be increased due to heat stress in several species including cattle, pigs and sheep [92,93,94]. Creatinine along with methylhistidine is a known indicator of muscle breakdown [95], the concentration of 1-Methylhistidine was also significantly increased in the female group. This indicates that the animals might be experiencing tissue break-down due to heat stress, the reason for such breakdown is not clear, however it could probably be due to increased protein catabolism, which is required for utilizing the carbon in amino acids for gluconeogenesis [96]. Methionine is a sulfur containing amino acid, and along with creatinine is involved in cellular antioxidant mechanism and is found abundantly on the surface of proteins exposed to very high oxidant fluxes [97,98]. The concentration of Pyruvate, an anti-oxidant [99] has been found to be increased under hyperthermia in pigs [100]. Pyruvate is also the precursor to alanine via alanine aminotransferase, and the entry of pyruvate into the TCA cycle through the pyruvate dehydrogenase complex is impaired due to heat stress, possibly due to the inactivation of PDH (Pyruvate dehydrogenase complex) complex due to the HS induced oxidative chain reactions generated by intracellular ROS (reactive oxygen species) [101]. Along with increased plasma concentration of Pyruvate due to the inactivation of PDH, the increased expression of *PDK4* (Pyruvate Dehydrogenase Kinase 4) is suggested to serve as a mechanism to reduce substrate oxidation and mitochondrial ROS production to protect HS induced cellular damage [6]. The expression of *PDK4* was significantly increased in the male group. Glycine stimulates protein synthesis, and has been found to inhibit oxidative stress in pig small intestine, the plasma concentration of glycine in the female group increased post HS while it reduced in the male group [102]. Similarly Alanine and Citrulline have protective effect against oxidative damage [103]. The difference in the plasma concentration of several of the above discussed metabolites together with increased expression of *PDK4*, *HMOX1* and down-regulation of *SOD1* indicates that the male group could have experience heat stress induced oxidative stress.

Several plasma metabolites were also oppositely enriched in the male and female groups. The plasma concentration of Tryptophan, 1-Methylhistidine, Acetate, Glycine, Aminoadipic acid, Alanine, Arginine, Citrulline, Cystathionine, Glutamate, Threonine, and Valine were all increased in the female group, while their concentration was reduced in the male group. Tryptophan metabolites are key neurotransmitters, that regulate immune response, increased plasma concentration of tryptophan could indicate intracellular protein degradation, and were found to increase the expression of apoptosis initiators in pig [104,105]. The plasma concentration of metabolites like glutamate, cystathionine and threonine are all associated with apoptosis and autophagy [106,107,108]. Several anti and pro apoptotic genes were also amongst the DEGs. This along with previously known effects of HS on protein denaturation and the proteotoxic effect of HS [16], and the increased concentration of amino acids that are by products of protein degradation suggest that HS induced cellular damage must have occurred in female group.

## 5. Conclusions

In conclusion, heat stress triggered a dynamic response in pigs; however, the response to heat stress was sex-specific. The reason for this sexual dimorphic response is not clear; however, evidence from other studies suggests that it could be due to the anatomical, physiological and hormonal differences. Future transcriptome and metabolome studies, along with blood parameters and hormonal analyses could provide more understanding about the effect of sex on the heat stress response in pigs. The results of this study along with increasing our understanding of porcine heat stress response will serve as a good reference for future studies. However, there are some limitations in this study, such as only six animals being used in this study for both transcriptome and metabolome analyses, and since only NMR was used for metabolite quantification, it resulted in limited detection of metabolites (only 48 metabolites) resulting in only a limited understanding of the effect of HS on metabolite accumulation, so complementing NMR analysis with mass spectrometric analysis might result in a more comprehensive understanding of the effect of HS on metabolite accumulation.

## Figures and Tables

**Table 1:** Composition of the total mixed ration (TMR) used during the experimental period.

Raw Material	Percentage
Corn	55.8
Soybean meal product	24.4
Wheat Bran	9
Soybean Hull	3
Molasses	3
Soybean Oil	2
Limestone	1.1
Lysine	0.4
Salt	0.4
Globik SW	0.3
TCP	0.4
Methionine-50	0.2
**Nutrient**	
Calcium	0.63
Total Phosphorus	0.5
Crude Protein	18
Crude Fat	4.9
Crude Fiber	4.6
Crude Ash	4.4
DRY MATTER	87.5
Arginine	1.16
Lysine	1.37
Methionine + Cysteine	0.7
D.Energy	3450 Kcal/kg

## Supplementary Materials

The following are available online at https://www.mdpi.com/2073-4425/11/5/540/s1. File S1: List of primers used in the q-RT PCR analysis. File S2: Summary of the mapping and alignment statistics. File S3: List of DEGs identified in response to heat stress. File S4: List of metabolites found to be differentially enriched in response to heat stress. Figure S1: PCA using RNA-Seq and metabolome data from all samples. Table S1: Summary of the measurements of average daily feed and water intake.
