# Supplementary material for: Genome-Wide Transcriptome and Metabolome Analyses Provide Novel Insights and Suggest a Sex-Specific Response to Heat Stress in Pigs
Source: Genes (Basel). 2020 May 11;11(5):540. doi: 10.3390/genes11050540 (PMC7291089; doi:10.3390/genes11050540)
Supplement: Supplementary file 1 [file genes-11-00540-s001.zip › Supplemetary_Table_ST1.docx]

|  | **Female** | |  | **Male** | |  | **pValue** | |
| --- | --- | --- | --- | --- | --- | --- | --- | --- |
| **CONDITION** | **25℃_60%** | **33℃_60%** | **Change Post HS** | **25℃_60%** | **33℃_60%** | **Change Post HS** | **SEX** | **TREAT** |
| **TREAT** | **Control** | **Treatment** | **∆** | **Control** | **Treatment** | **∆** |  |  |
| Average Daily Feed Intake (kg/day) | 2.50 | 1.41 | -1.09 | 2.84 | 1.69 | -1.15 | 0.628 | 0.012 |
| Average Daily Water intake (L/day) | 7.77 | 20.75 | 12.98 | 12.90 | 22.67 | 9.77 | 0.049 | 0.099 |

Table 1 : Summary of the measurements of Feed and water intake of the animals.
