# Supplementary figures and images for: Genome-Wide Transcriptome and Metabolome Analyses Provide Novel Insights and Suggest a Sex-Specific Response to Heat Stress in Pigs
Source: Genes (Basel). 2020 May 11;11(5):540. doi: 10.3390/genes11050540 (PMC7291089; doi:10.3390/genes11050540)

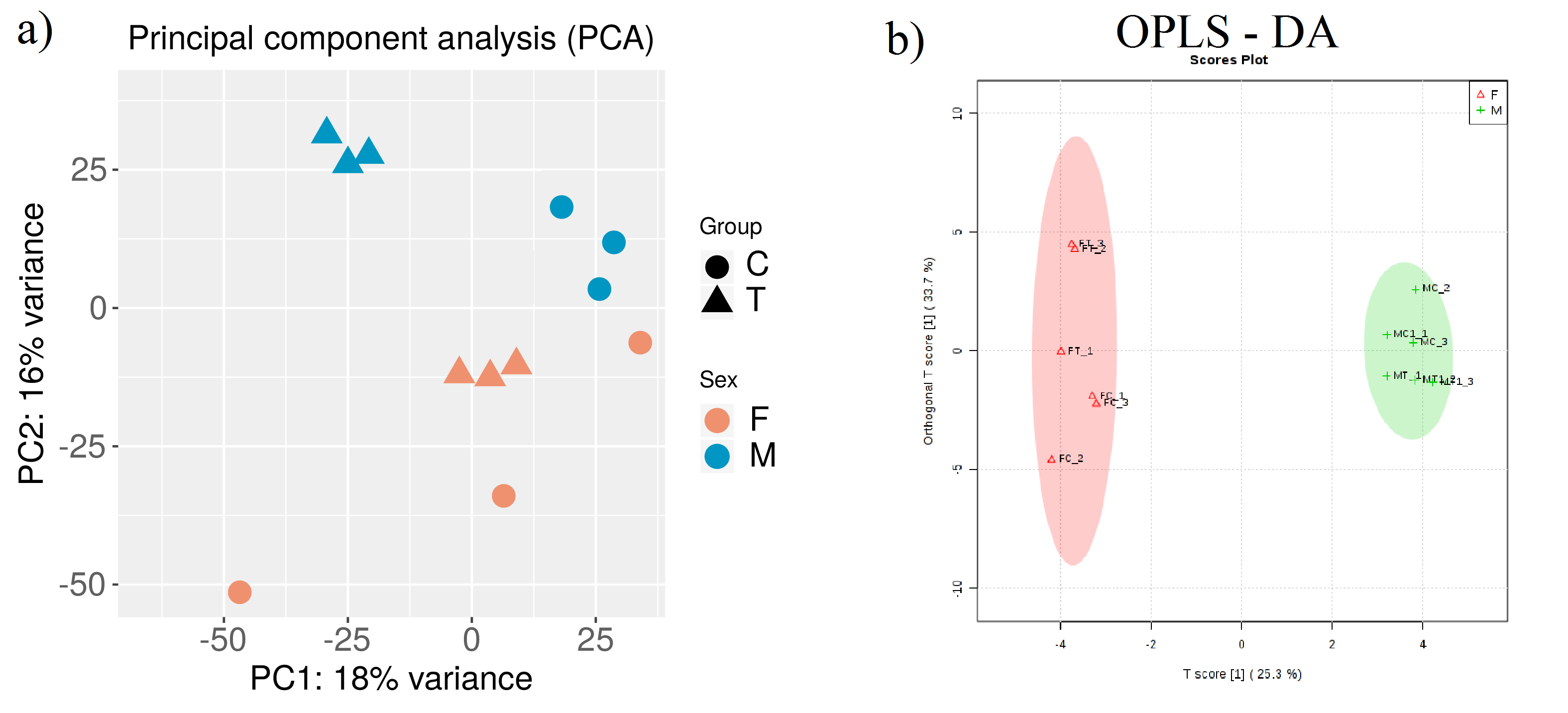

Supplement: Supplementary file 1 [file genes-11-00540-s001.zip › Supplementary_Figure1.jpeg]
